# Two mosaic terminal inverted duplications arising post-zygotically: Evidence for possible formation of neo-telomeres

**DOI:** 10.1186/1475-9268-7-1

**Published:** 2008-03-10

**Authors:** Art Daniel, Luke St Heaps, Dianne Sylvester, Sara Diaz, Gregory Peters

**Affiliations:** 1 Department of Cytogenetics, Western Sydney Genetics Program, The Children's Hospital at Westmead, NSW 2145, Australia

## Abstract

**Objective:**

To elucidate the structure of terminal inverted duplications and to investigate potential mechanisms of formation in two cases where there was mosaicism with cells of apparently normal karyotype.

**Results:**

A karyotype [46,XY,inv dup(4)(p16.3p15.1)/46,XY] performed on blood lymphocytes from a patient referred for developmental delay (case 1) demonstrated a normal karyotype in 60% of cells with a terminal inverted duplication 4p in the remainder. In case 2, referred for multiple fetal anomalies on an ultrasound scan, 33% of amniocyte colonies were karyotypically normal, with a terminal inv dup 10p in the remainder [46,XX,inv dup(10)(p15.3p11)/46,XX]. Duplicated FISH signals for GATA3 and NEBL loci (in case 2), and for the Wolf-Hirschhorn locus (case 1) confirmed the inverted structure of both duplications. In the GTL banded normal cells from both cases, there was a cryptic deletion detected by FISH of one copy of the subtelomere 4p (case 1, probe GS-36P21), and subtelomere 10p (case 2, probe GS-306F7). At pter on both inv dup chromosomes there was no FISH signal present for the specific subtelomere probe. However, a positive pantelomeric probe signal was detected at 4 pter and 10 pter in both the cryptically-deleted chromosomes and the inv dup chromosomes in the respective cell lines of both cases.

**Conclusion:**

An inv dup structure was evident for both cases on GTL bands, and confirmed by the various FISH studies. The presence of telomere (TTAGGG repeat) sequences at pter on the inv dup chromosomes (where more proximal chromosome specific subtelomeric probes were negative) was indicated by the pantelomeric probe signals in both cases. We conclude the most likely mechanism of origin in both cases was by sub-telomeric breakage in the zygote at pter, and delayed repair/rearrangement until after one or more subsequent mitotic divisions. In these divisions, at least one breakage-fusion-bridge cycle occurred, to produce inverted duplications. It is proposed then that two differently "repaired" daughter cells proliferated in parallel. In one daughter cell line (with an overtly normal karyotype) there was deletion of the subtelomere and presumed repair through capping by a neo-telomere (i.e. "healing", as initially proposed by McClintock). This occurred in both cases presented. In the other daughter cell of each case, it is proposed that chromosome stabilization was achieved (after replication) by sister chromatid reunion to form a dicentric, which broke at a subsequent anaphase, to form an inverted duplication (with loss of the reciprocal product, and the other daughter cell line). One inv dup was repaired without an interstitial specific subtelomere (case 1) and one was repaired with a duplicated specific interstitial subtelomere (case 2). After repair TTAGGG repeats were detected by FISH at each respective new pter.

## Background

Of the various types of inverted duplications (inv dup), most are non-mosaic, and one of the most frequently reported types involves an additional bisatellited inv dup(15) [1]. The inv dup(15), and similar types involving other chromosomes, arise during meiosis [2]. Similarly the interstitial direct and inverted duplications are also non-mosaic and have specific meiotic origins [3] unrelated to the present structures. The "mosaic inverted duplications", are a group derived by different mechanisms for which various postzygotic origins have been proposed by several authors [4-6]. The duplication in these cases often ends terminally on the chromosome arm with the former pter or qter region rearranged to an interstitial position. It has been proposed that a new chromosome telomere then has to be formed, or "captured" to stabilize the chromosome [6].

It was McClintock who originally proposed that telomeres could be "healed" after the breakage-fusion-bridge (BFB) cycle [7]. Such new chromosome telomeres can be synthesised directly onto non-telomeric DNA by telomerase [8], or in some cancer cells by ALT, the alternative (ALT) mechanism for telomere formation [9]. These newly synthesized telomeres are not detected by specific subtelomeric probes but are detected by probes for pantelomeric sequences.

These particular inv dup with the duplication ending terminally on the chromosome have been named "terminal inv dup" [10,11]. Such rearrangements with proven terminal inv dup structure include those described in chromosome 3p [12]; chromosome 4p [11,13,14]; chromosome 7q [10,15]; chromosome 8p [5,10,16]; chromosome 9p [6]; chromosome 10p [10]; and chromosome 10q [10]. Many of these terminal inv dup cases have been described as non-mosaic, and meiotic mechanisms of origin have been proposed to account for them [10,11]. For the mosaic cases another possible mechanism of origin has been proposed, whereby the initial event is formation of a dicentric by sister chromatid reunion in meiosis, transmission of the dicentric to the zygote, with postzygotic breakage to form the mosaic cell lines with different but related karyotypes [5]. In the present study two terminal inverted duplications were investigated to elucidate their structure and determine their most likely mechanism of origin.

## Results

**Figure 1:**
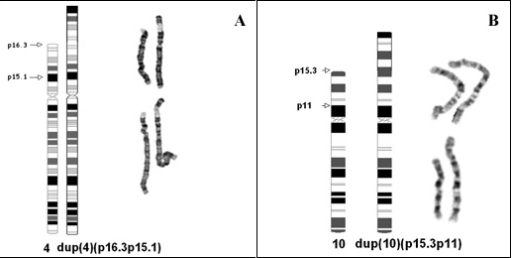
**A-B Title: Partial GTL karyotypes and ideograms of the two mosaic inv dup cases.** 1A: Case 1 with karyotype 46,XY,inv dup del(4)(:p15.1->p16.3::p16.3::p16.3->qter).ish inv dup del(4)(GS-36p21-,WSCR++,GS-963K6+,wcp4+)/46,XY.ish del(4)(p16.3p16.3)(GS-36p21-). 1B: Case 2 with karyotype 46,XX,inv dup(10)(:p11->p15.3::p15.3->qter).ish inv dup dup(10)(NEBL1+,GATA3+,GS-306F7++,GATA3+,NEBL1+,GS137E24+,wcp10+)/46,XX.ish del(10)(p16.3p16.3)(GS-306F7-,GS137E24+. The GTL patterns for both cases suggest an inverted duplication. Note in both cases a prior interstitial region is redirected to a telomeric position on the tip of the p-arm. In the rearranged chromosomes, the new pter regions should, in theory, be stabilised with telomeric sequences either by telomere capture or by neotelomere formation.

**Figure 2:**
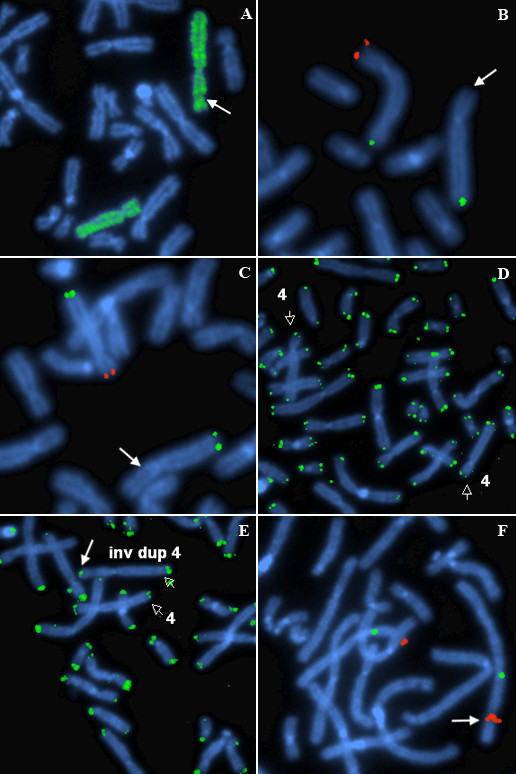
**A-F Structure and FISH results of the mosaic inv dup(4) from case 1**. 2A: a metaphase using FISH for WCP4 shows (green signals) that the extra material on 4p is indeed of intrachromosomal 4 origin. The newly interstitial sub-telomeric region in the (arrowed) inv dup (4) is poorly hybridized here, due to the presence of repetitive telomeric sequences (competitively excluded in the WCP hybridization). 2B: FISH on a metaphase from the apparently normal cell line showing a deletion of the sub-telomere probe for 4p (clone GS-36p21, shown in red) in one homologue (arrowed). The intact sub-telomere probe for 4q (clone GS-963K6 shown in green) is also demonstrated on both chromosomes. 2C: There is a sub-telomere deletion (absence of clone GS36p21 – shown in red) also in the interstitially located p-telomeric region of the (arrowed) inv dup(4). 2D: the pantelomeric probe for TTAGGG (green) is found on the 4pter of the chromosome, albeit with a missing subtelomere 4p locus, i.e. a positive TTAGGG signal is present on both (open arrows) the normal chromosome 4 and the 4p with the cryptic subtelomere deletion. In all FISH figures, chromosomes were identified by reversing the DAPI image (not shown). 2E: the pantelomeric probe (green) shows that TTAGGG repeats are found on the "new" 4pter (solid arrow) of the inv dup(4) chromosome (both normal 4 and inv dup(4) are indicated by open arrows). 2F: interstitially, there is a duplication (arrowed) of the Wolf-Hirschhorn locus (clone WHSCR in red) in the inv dup(4), confirming the inv dup structure. The CEP4 (4 centromere) control probe is shown in green.

**Figure 3:**
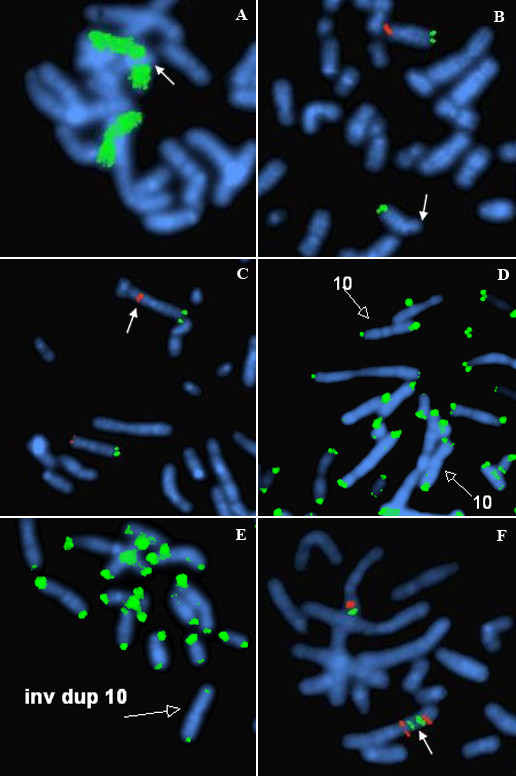
**A-F Structure and FISH results of the mosaic inv dup(10) from case 2**. 3A: FISH with WCP10 probe (green) with the (arrowed) inv dup(10) showing that the extra material is of chromosome 10 origin. Note the mid portion of the short-arm where the telomere to telomere join is located is poorly hybridized by the WCP10, possibly due to the presence of repetitive sub-telomeric sequences (competitively excluded in the WCP hybridization). 3B: FISH for the sub-telomere probe on the apparently normal cell line shows a (arrowed) sub-telomere 10p deletion (clone GS-306F7 shown in red). The intact 10q sub-telomere probe (clone GS-137E24 in green) is also demonstrated on both chromosomes. 3C: FISH for the sub-telomere probe 10p (clone GS-306F7 in green) showing a interstitial duplication (arrowed) of the 10p sub-telomere probe in the inv dup(10). The normal chromosome 10 (lower) exhibits normal probe signals at each p/q subtelomere. 3D: the pantelomeric probe (green) on the apparently normal cell line shows that TTAGGG repeats are found on the novel 10pter of the chromosome, which has a missing subtelomere 10p locus, i.e. a positive pantelomeric probe signal is present on both (open arrows) the normal chromosome 10 and the 10p with the cryptic subtelomere deletion. 3E: the pantelomeric probe (Oncor) on the inv dup(10) cell line shows that TTAGGG repeats are found on the new 10pter of the (open arrow) inv dup(10) chromosome. In this cell line the normal 10 also had a positive 10p signal for TTAGGG (not shown). 3F: FISH on an abnormal metaphase with GATA3 (shown in green and mapping to 10p14) and for NEBL (shown in red and mapping to 10p12.3) shows an inv dup pattern (arrowed), confirming the inv dup(10) structure.

GTL banded cells showed an inv dup structure (Fig 1). Case 1 with karyotype 46,XY,inv dup(4)(p16.3p15.1)/46,XY and Case 2 with karyotype 46,XX,inv dup(10)(p15.3p11)/46,XX. Cells of apparently normal karyotype were present in 60.0% of the cells from case 1, with the inv dup(4p) in the remainder. In case 2 there were 33.0% apparently normal cells, with the inv dup(10p) in the remainder. In the apparently normal cells of both cases (Figs 2B, 3B), there was a submicroscopic deletion of the subtelomere involved in the inv dup formation; thus each apparently "normal" cell line had a cryptic abnormality related to the inverted duplication event. In case 1, the 4p subtelomeric (ST) signal (for probe GS-36P21), was completely missing in the inv dup chromosome (Fig 2C). It was also missing in one 4p of the apparently normal cell line. In case (2), there was a duplicated interstitial ST signal (for probe GS-306F7) in the inv dup(10p), (Fig 3C), with no 10p-specific subtelomeric signal at pter. Here also, there was a deleted sub-telomeric signal in one 10p of the apparently normal line (Fig 3B). Duplicated signals for GATA3 and NEBL loci (case 2, Fig 3F), and for the Wolf-Hirschhorn locus (case 1, Fig 2F) confirmed the inv dup structure of both duplications. In the normal and inv dup cells of both cases, p-terminal regions of chromosomes 4, 10 (and all other chromosomes) showed normal signals with the pantelomere probe. This finding suggest possible formation of neo-telomeres for both present cases 1 and 2. In neither case was there evidence from MLPA for telomere captures or unbalanced translocations involving telomeres.

## Discussion

### Hypotheses of terminal inv dup origin

One widely accepted hypothesis for the origin of these terminal inv dup mosaics has been that a post-zygotic unequal sister chromatid exchange has occurred [4,14]. On this basis, the pter sub-telomere of the apparently normal cell line would be derived by unequal exchange from the homologue of the chromosome in question. However, there are several cases where this is not the situation and where the apparently normal cell line or the inv dup cell line has a neo-telomere (both present cases) or a captured sub-telomere from a non-homologue [6]. These findings rule out this hypothesis of origin in some of the cases at least. In the present cases, both apparently normal cell lines had a likely neo-telomere adjacent to a deleted chromosome specific sub-telomere, ruling out the Kotzot unequal crossing-over hypothesis. Therefore, alternative mechanisms should be considered.

### Evidence for multiple events in the origin of these mosaic terminal inverted duplications

Two studies have proposed a mechanism for the origin of these terminal inv dup involving a delay between zygote formation and generation of the mosaicism by the invoking of different repair options. In the first hypothesis, [5], meiotic breakage and sister chromatid reunion produces a dicentric which is inherited intact by the zygote. Postzygotic breakage then gives rise to the mosaic cell lines with different karyotypes via the dicentric breakage fusion bridge cycle. Since the sister product of the duplicated dicentric breaking close to one of the centromeres in the distal duplicated segment would be a chromosome with a near complete deletion of 10p or 4p, neither of which were detected, this interpretation seems implausible.

Whereas this hypothesis may explain the existence of the odd structure of a terminal inv dup in a non-mosaic case, it doesn't explain how mosaicism with the cell lines containing near normal 10p or 4p (but actually with deleted subtelomeres) could arise.

In the second hypothesis, that of Chabchoub et al [6] a double strand chromosome break in the zygote is proposed to occur followed by mitotic separation of the broken sister chromatids (while the chromosome repair is delayed) during which the break is thought to have been unresolved or non-stabilised. One of the chromatids in their case was shown to acquire a telomere by neo-telomere formation giving rise to an apparently normal cell line, which was proven to also have a deleted chromosome specific sub-telomere. The other chromatid formed an inv dup and was subsequently healed by telomere capture from a non-homologous chromosome.

In these last few steps, dicentric formation by sister reunion; breakage and loss of the fragments distal to the terminal inv dup; are implicit but cannot be observed in retrospect. However, this model can explain the cryptic ST deletion in the near normal cell line (unlike the Pramparo model) and seems to fit the situation in both of our cases, i.e. a delay in the healing of a chromosome break with different destinies for the two chromatids involved when they segregate to daughter cells.

There is evidence for multiple events (with separate destinies arising from a shared initial event) in both cases of the present study. In this connection, the apparently normal cell line in case 2 [inv dup (10p)] is actually not normal and has a complete deletion of the sub-telomere 10p whereas the complementary inv dup(10p) has an interstitial duplication of the 10p subtelomere at the interstitial point of the duplication and a neotelomere at the p-terminus. The absence of the subtelomere in the near normal cell line with a normal ST locus in the inv dup cell line is not simple to explain. However some mechanisms of DNA repair [17] of double strand breaks (e.g. non-homologous end joining) can create a deletion if sequences surrounding a lesion are lost whereas other mechanisms of repairing double strand breaks (e.g. homologous recombination – HR) utilise a template from a homologous chromosome or sister chromatid which is copied and the sequences can be completely restored.

If such different repair mechanisms were invoked in the different daughter cell lines of this case from the same initial break in a parent cell then a deleted chromosome and a restored subtelomere locus may co-exist in the different daughter cells involved. The cell line resulting finally in the inv dup could have been repaired by a mechanism such as HR with prompt sister chromatid reunion (and dicentric isochromosome formation) without a deletion of the subtelomere.

Alternatively, if BFB cycles are involved, in case 2 the presence of a duplicated subtelomere in the inv dup chromosome merely reflects that the p-arm sub-telomere FISH probe was proximal to the initial sub-telomere break and hence was retained, for at least one of the possible BFB cycles. In another BFB cycle, a secondary break could have occurred proximal to this probe locus, deleting it from the overtly normal breakage product. Applying this argument to case 1, the available evidence is consistent with a primary chromatid break proximal to the subtelomere probe locus, effectively eliminating it from all possible BFB products. In this connection it may be relevant that the subtelomere probe used for 4p is more distally located than that for 10p.

Case 1 also fits the Chabchoub hypothesis but the evidence for different destinies from an initial shared event is not so clear since both "normal" cell line and terminal inv dup cell line have a deleted original subtelomere for which there is no evidence either at the breakpoint of formation in the middle of the 4p+ inv dup chromosome or on the subtelomere of the 4p in the cryptically deleted cell line. The Chabchoub hypothesis infers that a chromosome break can remain unrepaired for at least 1–2 cell divisions.

It is reasonable to ask whether there is any evidence for such delayed repair in other chromosome rearrangement types since a broken chromosome/DNA molecule is known to be unstable due to "sticky ends". A classic example of this "stickyness" is seen in specific cell lines or tumour types where telomeres have been reported to be very reduced or absent and chromosome end-to-end chains tend to form [18].

### Evidence from other cytogenetic phenomena for delayed repair events when chromosome breakage occurs

There are relatively common mosaics that illustrate the principal that delayed repair events following chromosome breaks do occur and that different daughter cell destinies can arise from the same initial break. Some of these are the mosaics of isochromosomes involving human acrocentrics and are typified by the cases where one cell line has a p-arm deletion and the other cell line has an isochromosome. Such acrocentric cases have been reviewed in Tuerlings et al, [19]. Another group of examples is the various mosaics seen in isochromosomes (18p) and/or an i(18q) [20,21]. Some of the so called "jumping translocations" exhibit similar phenomena where after breakage and delayed repair, different stabilising translocations may occur in different daughter cells [22]. Other jumping translocations have a different origin where rare unstable sequences are involved and re-breakage at or near the same site (rather than different cell progeny from a single original breakpoint) is responsible for the multiple rearrangements [23].

In summary, the stabilising of chromosome breaks in various types of chromosome rearrangements may not occur until a few cell cycles have passed. We suggest a similar phenomenon happens where there is a postzygotic origin and parallel development of cell lines for the terminal inv dup mosaics. In this connection there is evidence for a high proportion of mosaics [5] in these terminal inv dup and it is possible that this mechanism may explain the origin of this entire group.

## Conclusion

The two terminal inv dup of the present cases most probably arose by the same mechanism. i.e. possibly according to the hypothesis of Chabchoub et al [6]. In this proposal a double strand chromosome break occurs in the zygote followed by mitotic separation of the broken sister chromatids (while the repair is delayed) during which the break is though to have been unresolved or non-stabilised. One of the daughter chromatids segregating to a daughter cell subsequently acquires a new telomere by neo-telomere formation or telomere capture giving rise to an apparently normal cell line which, as we show, has a deleted chromosome-specific sub-telomere. The other chromatid forms an inv dup through breakage of a transient dicentric, and is subsequently healed by neo-telomere formation or by telomere capture from a non-homologous chromosome. Alternatively, the breakage-fusion-bridge cycle of McClintock may have produced both cell lines similarly after a delay of one or more cell divisions. In the present cases it appears possible that neo-telomere formation was involved in the repair and stabilization of both cell lines of both cases.

## Materials and Methods

Case 1 was detected in the lymphocyte culture of a 3 year old boy referred for karyotyping because of minor non-specific dysmorphisms and mild intellectual handicap suggesting Fragile X syndrome. An EDTA specimen was sent in parallel for Fra(X) testing which was negative.

Case 2 was detected in an in situ amniocyte culture of a female fetus referred at 14 weeks, 3 days gestation because of abnormalities detected on an ultrasound scan where the crown-rump length was 9.2 cms. These abnormalities were bilateral talipes and rocker-bottom feet, right renal pelvis dilatation, and bilateral cleft lip and palate extending the full length of the palate. The fetal head, thoracic cavity, abdominal cavity, spine, genitalia, and heart were not assessed in this scan. After being counseled on the ultrasound findings and the prenatal cytogenetics report, the parents decided on a termination of pregnancy following which fetal autopsy was not performed. Initially DNA was extracted and archived on a white blood cell pellet from case 1 and cultured amniocytes from case 2. This DNA was used for MLPA for subtelomeric sequences.

Both cases were banded with routine high resolution GTL techniques. Fluorescence in situ hybridization (FISH) was performed in these two mosaic cases with the respective directly labeled wcp, subtelomeric probe, and pantelomeric probe, to address the "apparently normal" cell line. This FISH probing was performed in addition to confirm that in the aneuploid cell line: the extra material was of intrachromosomal origin; to detect the location of the specific subtelomere; and to detect any neo-telomeres. The FISH for pantelomeres was performed in conjunction with MLPA for all sub-telomeres to exclude cryptic telomere capture events. In addition specific FISH probes known or suspected to be located in the duplicated segments were used. These included the Wolf-Hirschhorn probe (hybridizing to 4p16.3) for case 1; and GATA3 (hybridizing to 10p14) and NEBL (hybridizing to 10p12.3) probes for case 2 which were applied to detect the orientation of the duplicated segment.

### Choice and performance of FISH

FISH was performed by standard techniques. The telomeric specific clones used for FISH in the study were those documented [24] unless specified otherwise. These are used to identify all of the 41 unique sub-telomeric sequences. The identity of the specific subtelomere clones used in the study was for case 1, clone GS-36P21 (*Incyte Genomics*) for the 4p subtelomere, and clone GS-963K6 for the 4q subtelomere. For case 2, clone GS-306F7 was used for the 10p subtelomere and clone GS-137E24 for the 10q subtelomere. Critically the 4p clone (GS-36P21) maps from 55,628 to 207,197 bases from 4pter, whereas the 10p clone (GS-306F7) maps from 259,607 to 365, 076 from 10pter. The WCP probes for chromosomes 4 and 10 were from *CAMBIO*. The pantelomere probe (*CAMBIO*) was used to detect the presence of a neo-telomere where there was no detection of the increased presence of a specific subtelomere by MLPA. The specific probes used to address the duplicated segments were WHSCR (Vysis) for Case 1 and GATA3 (RP11-379F12 from The Australian Genome Research Facility (AGRF) and NEBL (RP11-56H7 from AGRF) for case 2.

### MLPA testing for subtelomere screen

The presence of telomere captures or unbalanced cryptic telomere translocations was tested for by a MLPA screen for all available subtelomeres (single probes for each). For this testing kit P070 (MRC Holland) was used. This was performed according to the methods and reagents described on the MRC-Holland website [25].

## Authors' contributions

AD designed the study, and drafted the manuscript. LH had oversight of the growing of FISH probes, labelling of probes, and probe hybridizations. DS performed FISH hybridizations and analysed FISH signals for case 1. SD performed FISH hybridizations and analysed FISH signals for case 2. GP was the co-designer of the study, was involved in discussions of the results and interpretation of the phenomena.
